# Targeted Muscle Reinnervation—an Up-to-Date Review: Evidence, Indications, and Technique

**DOI:** 10.1055/a-2521-2199

**Published:** 2025-04-01

**Authors:** Ava G. Chappell, Matthew D. Ramsey, Seong Park, Gregory A. Dumanian, Jason H. Ko

**Affiliations:** 1 Division of Plastic and Reconstructive Surgery, Department of Surgery, Northwestern University Feinberg School of Medicine, Chicago, Illinois

**Keywords:** targeted muscle reinnervation, nerve transfers, painful neuromas, myoelectric prosthesis

## Abstract

Targeted muscle reinnervation (TMR) is a surgical technique originally created to improve prosthetic function following upper extremity amputation. TMR has since been shown to be effective in the prevention and treatment of chronic postamputation phantom and residual limb pain in both upper and lower extremity amputees and for neurogenic pain in the nonamputee patient population. This article provides a current review of the various indications for TMR and surgical techniques, organized by amputation site, timing, and regional anatomy.

## Introduction

Targeted muscle reinnervation (TMR) was developed based on the principle of “giving a nerve somewhere to go and something to do,” which results in direct nerve-to-nerve healing. 1 Coaptation of severed peripheral nerves to freshly divided motor nerve branches enables the fascicles of the proximal nerve to grow into the motor end plates and reinnervate the target muscle rather than forming a symptomatic neuroma. 2 3 4 The injured/transected peripheral nerves retain their upper nerve innervation from the motor cortex, necessitating the absence of upper motor nerve injury for effective TMR. This reinnervated muscle also acts as a bioamplifier that can be detected on an electromyogram. Thus, as first performed by Souza et al in 2014, TMR was originally intended to improve myoelectric prosthesis control for amputees. 4 The “secret” of TMR is that the donor motor nerve does not become a symptomatic neuroma and the TMR nerve transfers “steal” the end receptors in the target muscle. While divided motor nerves form neuromas, there are no case reports in the literature of a symptomatic motor nerve neuroma.

Chronic neuroma pain is often described as a sharp, electric, burning pain associated with minor stimuli (e.g., light touch, pressure, temperature changes) or no stimulation. 5 The neuromas can develop due to a nerve injury following trauma, amputation, or surgery as the injured mixed (motor and sensory) or sensory nerve sprouts in a chaotic manner to find a distal nerve or end organ in an attempt to heal. 6 7 8 With amputation, however, there are multiple severed nerves without any distal target organ to reinnervate, resulting in uncontrolled proliferation of axons and connective tissue, forming a neuroma. 9 Approximately 25 to 71% of these neuromas are symptomatic. 10 11 12 13 14 The symptomatic neuromas are the primary cause of postamputation pain, including residual limb pain (RLP) and phantom limb pain (PLP; pain or unpleasant sensation referred to the missing limb; possibly due to the interaction between neuroma and multiple levels of the central nervous system [CNS]; correlated with cortical reorganization and gray matter changes). 13 15 16 17 18 19 20 21 22 23 The symptomatic neuromas not only affect patients' quality of life but also their functional abilities and independence as the neuroma pain can be exacerbated by prosthesis use, making it difficult or even intolerable to wear. 11 24 25 26 There are approximately 2 million amputees living in the United States, and this population will increase to 3.6 million by 2050 with approximately 185,000 major limb amputations being performed each year. 26 27 Considering that at least 25% of the amputee population will develop chronic RLP and/or PLP due to symptomatic neuromas, chronic neuropathic pain is a significant public health issue. 11 13 14

## Evidence

### Myoelectric Prostheses Control

The original case series from Kuiken et al (2004, 2007) demonstrated the benefits of TMR for myoelectric prosthesis control in the proximal upper extremity amputee. 2 3 Since those studies were reported, more have followed that support the benefits of TMR for myoelectric prosthesis control following upper extremity amputation. 4 28 29 30 TMR facilitates intuitive prosthesis control and allows two prosthetic joints to be moved simultaneously. In addition, it enables control sites for multiple prosthetic functions (hand open/close, elbow flexion/extension, wrist flexion/extension, and wrist prono-supination). 31 After a decade of performing TMR for prosthetic control, it was observed that TMR may also treat chronic postamputation (both neuropathic and phantom) pain. 10

### Phantom Limb Pain and Neuroma Pain

While PLP is distinct from symptomatic neuroma pain, these pain sources are not always mutually exclusive, and, currently, there is no widely accepted standard to treat phantom limb or neuroma pain. However, various surgical strategies (e.g., neuroma excision and burying into nearby muscle, bone, nerve, back on itself; regenerative peripheral nerve interfaces [RPNIs]) and nonsurgical strategies (e.g., pharmacologic therapy, psychological and behavioral strategies, and interventional/minimally invasive procedures) exist to treat PLP and/or symptomatic neuroma pain. 5 26 32 33 34 35 36 37 38 39 40 41 42 43 44 45 46 47 48 49 50 A recent meta-analysis reports that surgical procedures resulted in a substantial decrease in neuropathic pain in 77% of the patients, without significant differences between surgical techniques (“excision and transposition, excision only, excision and repair, neurolysis and coverage, and excision and cap”). 51 While neuromodulators, specifically gabapentin, have been shown to have positive effects in preventing postamputation PLP in pediatric patients, 52 benefits to medical and pharmacological treatments in treating PLP are still inconclusive. 50 53 54

While there may not be a “gold standard,” recent studies demonstrate the promising role of TMR in both the prevention and treatment of phantom limb and neuroma pain. A prospective, single-blind, randomized control trial study comparing TMR to standard surgical strategies (neuroma excision and muscle burying) in 28 amputees (total of 30 limbs) with chronic neuroma-related RLP and PLP demonstrated that there was a trend in a greater reduction in numerical rating scale (NRS) with worst PLP and RLP at 1-year follow-up in the TMR group versus standard; however, the percentage of patients reporting no pain or mild pain at 1.5-year follow-up was significantly higher in TMR group than in standard treatment group for both RLP (67% vs. 27%) and PLP (72% vs. 40%). 1 Similar to these results, a prospective case series on 33 amputees who underwent TMR for neuroma pain after being ineligible for randomization, or refusal to be randomized, reported improvement in limb function and a significant decrease in the numeric pain score on 11-point NRS by 1 year after TMR for both RLP (from 6.4 ± 2.6 to 3.6 ± 2.2; mean difference of −2.7) and PLP (6.0 ± 3.1 to 3.6 ± 2.9; mean difference −2.4). 55 The positive impact of TMR in treating neuropathic and PLP, as supported in the studies above, may be due to its effects on peripheral nerve regeneration, which has been reflected in histology studies showing the restoration of axon count, size, and myelination after TMR in a rabbit amputation model, electromyography studies showing synaptic inputs to reinnervated muscles, and MRI studies suggesting the role of TMR in reversing pathological cortical reorganization associated with PLP. 56 57 58 59 60 61 62

TMR can be used at the time of amputation for the prevention of symptomatic neuromas and PLP (acute TMR) or in an established amputee for the treatment of chronic neuroma pain (delayed TMR). 63 Numerous studies to date have shown a reduction in neuroma pain without the formation of new neuromas and enhancement of myoelectric prosthesis use after acute TMR and delayed TMR. 3 4 10 34 64 65 66 67 68 69 A recent cohort study comparing 100 patients undergoing below-knee amputation (BKA) with acute TMR versus 100 patients undergoing BKA and traction neurectomy and muscle implantation demonstrated significantly less RLP and PLP in the TMR cohort. 70

The benefits of TMR can be extended to nonamputees with symptomatic neuromas or unreconstructable nerve injuries with multiple studies showing improved pain, function, and quality of life outcomes in these patients treated with TMR. 71 72 73 74 75 In addition, successful reinnervation following TMR was observed in 96% (94/98) of transferred nerves, 6 suggesting the potential usage of TMR as a first-line surgical treatment option for symptomatic neuroma pain amputees and nonamputees alike. While sacrificing a motor branch in a nonamputee may be questionable, however, TMR involves the use of only redundant motor branches to minimize downgrading of motor function following nerve transfer. Evidence to support this concept includes a 15-patient case series of nonamputees treated with TMR for symptomatic neuromas, no patients had postoperative motor weakness of their donor nerve. 75 The ability of TMR to give transected nerve endings “somewhere to go and something to do,” and potentially improve organized peripheral nerve regeneration, is the main concept behind its efficacy in treating mixed and sensory peripheral neuromas in amputees and nonamputees. 1 76

## Preoperative Considerations

### Anesthesia (Authors' Preference)

Procedures are performed under regional anesthesia for distal amputations, and under general anesthesia for more proximal procedures. Long-acting muscle relaxants and local anesthetics are avoided to permit motor nerve identification with nerve stimulators. Regional anesthesia is a good consideration for patients in the supine position who have significant comorbidities. Tourniquet time for upper and lower extremity TMR should be less than 45 minutes to allow for reliable intraoperative stimulation of motor nerves. 76

### Surgical Techniques (Authors' Preference)

Prior to the operation, the locations of the painful neuromas (if present) are marked, as they are often heralded by Tinel's signs. The essential operative steps of TMR are (1) symptomatic neuroma identification and excision when feasible, (2) preparation of neuroma stump to healthy fascicles, (3) recipient motor nerve identification with a nerve stimulator, and (4) tension-free coaptation. Recipient motor nerves are redundant branches of local muscles. The motor nerve must be redundant to *not* lead to appreciable loss of function when transected to serve as a recipient for the donor nerve ending. Typically, the donor nerve is coapted as close as possible to the target muscle to reduce time to reinnervation. 77 The coaptation is performed under loupe magnification with 6–0 or 7–0 polypropylene epineural sutures. Size mismatch is common and not of concern.

## Acute Targeted Muscle Reinnervation

Given the high-level evidence of TMR for both improved myoelectric prosthetic use and RLP/PLP reduction, acute TMR (commonly defined as within 14 days/2 weeks of major limb amputation) is now performed routinely. 1 34 67 68 70 76 78 In fact, data suggest that TMR performed at the time of amputation provides greater RLP and PLP relief than no TMR performed acutely. 34 Complicating considerations include the potential loss of muscle bulk and prosthetic padding with TMR nerve transfers and the finding that up to 25% of major limb amputees without any nerve interventions have painless limbs without phantoms. 13 Acute TMR risks overtreatment of the amputee but does so to provide improved comfort for the 25% of amputees that develop severe pain and phantoms. Acute TMR should be considered when (1) the nerves have not been avulsed proximally, (2) there are redundant motor targets, (3) there is well-vascularized soft tissue to close, and (4) patients can tolerate the additional time in the operating room with anesthesia ( **see** **Table 1** **for a summary of surgical approaches and nerve transfers for TMR in the acute setting** ). 68 70 76 79 80

**Table 1:** The surgical approach to the acute amputee is described with corresponding sensory and motor target nerves for coaptation and surgical tips for a successful operation

Amputation type	Incision/Dissection	Sensory/Mixed nerves	Target motor nerves	Prosthetic function	Notes
Shoulder disarticulation	Transverse, 2 cm inferior to clavicle split pectoralis heads and develop space can transect pectoralis minor if significant scar present	Median	Split sternal head	Hand function	Most variable of TMR patterns presence/absence of humeral head indicates pectoralis position long thoracic can be coapted to if listed targets absent/nonfunctional MCN and MN prioritized for elbow flexion and grasping
		Ulnar			
		Musculocutaneous	Clavicular head	Elbow flexion	
		Radial	Thoracodorsal	Extensors	
Transhumeral	Anterior, biceps raphe retract short head medially posterior, triceps raphe begin blunt dissection cephalad	Median	Short head biceps	Hand function	Mark biceps and triceps raphe preop adipofasical flap used to increase spatial differentiation preserve native innervation to long head of biceps and triceps for elbow function
		Ulnar	Brachialis		
		Distal radial	Lateral head triceps	Hand open	
Transradial	Large volar and dorsal fish-mouth incisions planned to prevent closure directly over terminal residuum	Median	Brahioradialis, FDS/FDP	Hand function	Minimum of 5 cm radius and/or ulnar for prosthetic variable transfer pattern depends on indication for amputation
		Ulnar	FCU		
		Sensory branches radial	Pronator quadratus, FDS/FDP, ECR	RLP/PLP prevention	
AKA	Posterior, vertical 10 cm mid-axis Bluntly dissect hamstring musculature medial thigh at level of Hunter's canal	Tibial	Semimembranosus	Plantarflexion	See cadaveric study for detailed motor entry points (Agnew et al 2012) intuitive powered prosthetics for stair climbing rely on TMR
		Common peroneal	Long head biceps femoris	Dorsiflexion	
		Posterior cutaneous nerve of thigh	Short head biceps femoris	Plantar sensation	
		Saphenous	Vastus lateralis	RLP/PLP prevention	
BKA	Within the operative field, supine	Tibial	Soleus, FDL	RLP/PLP prevention a	See cadaveric study for detailed motor entry points (Fracol et al 2018) 83
		Deep peroneal	Tibialis anterior	RLP/PLP prevention a	
		Common peroneal	Peroneus longus	RLP/PLP prevention a	

Abbreviations: AKA, above-knee amputation; BKA, below-knee amputation; ECR, extensor carpi radialis; FCU, flexor carpi ulnaris; FDL, flexor digitorum longus; FDS/FDP, flexor digitorum superficialis/profundus; MCN, musculocutaneous nerve; MN, median nerve; TMR, targeted muscle reinnervation.

a Prosthetics employing these myoelectric signals currently only exist in the laboratory setting.

### Shoulder Disarticulation

**Video 1** Delayed shoulder TMR for prosthesis optimization. TMR, targeted muscle reinnervation.


The approach to TMR in the setting of shoulder disarticulation is among the most difficult to perform given multiple factors. The first is that a damaged or compromised surrounding soft tissue envelope is very common, requiring a thorough preoperative evaluation. This evaluation assesses both the soft tissues that the prosthesis will rest on and the remaining musculature for potential motor targets. Volitional control of the pectoralis major and minor muscles and latissimus dorsi is required since these are the common motor targets at this level. Second, the presence or absence of a humeral head should be evaluated with plain radiographs. Absence indicates that the pectoralis major and its neurovascular bundle should be expected to have shifted 4 to 6 cm medially, while the presence of the humeral head may indicate a residual triceps with radial nerve (RN) innervation that may deserve preservation for an elbow extension signal. 78 81 A review of 26 consecutive TMR patients displays the complexity and variability in the pattern of nerve transfers for the shoulder disarticulation level. 10 In comparison from the same study, all 16 transhumeral patients received the same pattern of nerve transfers, to be discussed below, indicating reliable anatomy, whereas 10 shoulder disarticulation patients received 7 different transfer combinations. 10

Dissection begins with a transverse incision 2 cm below the clavicle and continues to identify and develop the potential space between the sternal head (SH) and clavicular head (CH) of the pectoralis major, which is often marked by a stripe of fibrofatty tissue. The CH overlaps the SH, and so the fibrous cleft is angled superiorly as the dissection deepens. The motor nerve to the CH accompanies the vascular pedicle and is located at the junction of the middle and lateral thirds of the clavicle. The motor nerves to the pectoralis can be described as a medial clump, a middle grouping, and a lateral fascicle that goes through the pectoralis minor to the lateral/inferior border of the muscle. It is crucial to locate all native motor nerves to the pectoralis major such that complete denervation can be achieved prior to nerve transfers. The brachial plexus cords are next identified deeper in the fibrofatty tissue between the SH and CH either medial or lateral to the pectoralis minor. In cases of secondary fibrosis from trauma, the pectoralis minor tendinous insertion on the coracoid process can be released. If more length is needed, the nerves can be mobilized to lie medial to the pectoralis minor. Depending on the patient, correct identification of cords can be quite challenging, however, postoperative myoelectric mapping solves this issue. Spatial differentiation and successful neurotization are the two primary goals. Ideally, the musculocutaneous nerve (MCN) is coapted to the motor nerve to the CH, median nerve (MN) and ulnar nerve (UN) coapted to split segments of the SH, and RN to the thoracodorsal nerve. 78 81

**Fig. 1:**
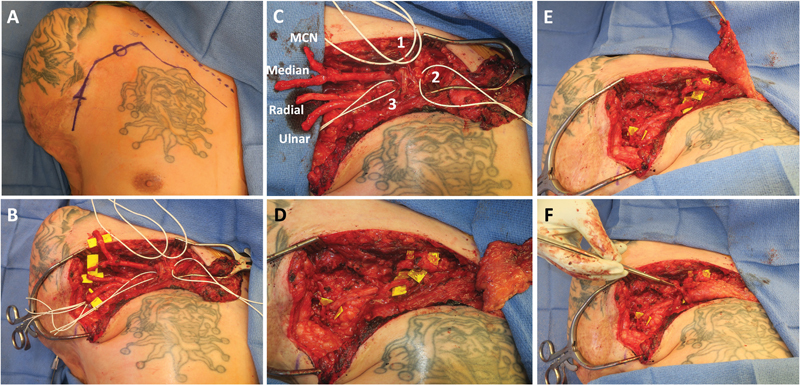
Example of delayed shoulder TMR for myoelectric prosthesis control. The patient is a 42-year-old male with a previous right upper extremity trauma complicated by infection requiring shoulder disarticulation who presented with no pain but poor myoelectric prosthetic control. Of note, a pedicled latissimus muscle flap was previously required for soft tissue coverage and preop findings were as follows: triceps 0/5, deltoid 1 to 2/5, remnant proximal biceps 2/5, latissimus muscle flap 2/5 (retained voluntary contracture). ( **A** ) demonstrates the planned incision two finger-breadths below the clavicle medial to sternal notch then laterally and inferiorly along deltopectoral groove. ( **B, C** ) demonstrate the nerves dissected and ready for transfer to motor targets with Video 1 displaying the target motor nerve being stimulated. ( **D** ) demonstrates the coaptations performed with 7–0 prolene in end-to-end epineural fashion: (1) MCN to the motor branch of the clavicular head of pec major (elbow flexion), (2) median nerve to lateral pectoral nerve (medial and upper sternal head of pec major muscle; hand close), (3) radial nerve to medial pectoral nerve (lower lateral pec major sternal head; elbow extension and finger/thumb extension), (4) ulnar nerve to thoracodorsal nerve (not shown in the figure). ( **E** ) A medially based, pedicled adipofascial flap was raised and inset ( **F** ) between the clavicular and sternal heads of the pec major to buffer signaling of elbow flexion (MCN to clavicular head) and elbow extension (radial to sternal head) for the surface electrodes. MCN, musculocutaneous nerve; TMR, targeted muscle reinnervation.

In the setting of delayed shoulder TMR for prosthesis optimization ( Fig. 1 and Video 1 ), it is recommended to surgically thin the skin overlying the pectoralis major to assist with signal recognition, although newer pattern recognition technologies may obviate the need for thinning. A rich subdermal plexus prevents skin flap necrosis or poor healing. The MCN and MN represent the two most important nerve transfers as they allow for elbow flexion of the device and hand closing. For this reason, the MCN is transferred to the CH as this achieves the most robust, reproducible myoelectric signal for the surface electrodes. 78 81

### Transhumeral

The goals of acute TMR in the transhumeral patient are to preserve native elbow flexion and extension signals via the MCN innervation of the long head of the biceps and RN innervation of the long head of the triceps, respectively, while creating novel “hand open” and “hand close” signals. Before anesthesia, preoperative Tinel's signs can often help identify end neuromas of the MN, UN, and RN. The biceps and triceps muscle bellies are also marked by an absence of a hand and humeral condyles can make locating intraoperative landmarks challenging. Positioning typically begins supine with an arm board for the anterior nerve transfers, and the patient is flipped to the prone position for a posterior approach. 78 81 Alternatively, transhumeral TMR can be performed in the lateral decubitus position to avoid a position change. For neuroma control only, all transhumeral TMR nerve transfers can be performed supine through a single anterior incision. 82

The longitudinal anterior incision is made along the midportion of the biceps. A proximally based adipofascial flap is created approximately 6 cm wide and dissected proximally until the tendinous origin of the long head of the biceps is visualized. The fibrous septum between the heads of the biceps is identified carefully. Blunt dissection in this septum reveals the motor nerves emanating from MCN to innervate the biceps muscle bellies. Proximally, the MCN branches to innervate the two heads, while distally it continues to innervate the brachialis (MCN-Br) and form the lateral antebrachial cutaneous (LABC) nerve. The motor branches to the two biceps heads enter the muscle approximately one-third the length of the humerus and enter the brachialis approximately two-thirds along its length. After identification of these entry points, dissection proceeds to identify the MN and UN. This is facilitated by transposing the short head medially and bluntly dissecting between the short head and the intermuscular septum (IMS). The MN will be identified adjacent to the brachial artery and anterior to the IMS and is transferred to the short head of the biceps while preserving the native MCN innervation of the long head. The UN will be identified posterior to the IMS and transferred to a motor branch to the brachialis. It must be noted that proximal branching of the MCN to the short head can be variable in number—all MN motor branches need to be identified and divided as they travel into the short head. The previously elevated adipofascial flap is inserted into the raphe to assist with spatial differentiation of the myoelectric signals and the incision is closed. 78 81

The posterior incision is also made along the previously marked midportion of the triceps muscle belly and proceeds analogously to the anterior approach with the development of an adipofascial flap. The muscle bellies are then bluntly dissected beginning cephalad near the inferior margin of the deltoid. Medial retraction of the long head typically reveals the RN. The branch to the long head is typically quite proximal and not encountered during dissection while multiple branches can be easily visualized innervating the lateral head in this space. The distal RN branch is then divided and coapted to the newly divided motor nerve of the lateral head; the adipofascial flap is inserted analogous to the anterior approach and the incision is closed. 78 81

### Transradial

Preoperative assessment must evaluate remaining forearm bony structures and nerve function. A minimum of 5 cm of residual radius and/or ulna is required to correctly fit a prosthesis, and nerve damage proximal to the elbow will decrease the chance of successful nerve transfers. The patient is positioned supine with an arm board and tourniquet. Typically, for a primary transradial amputation that is more proximal to the elbow, a large volar and dorsal fish-mouth-shaped incision is used. Full-thickness skin flaps are elevated on the volar and dorsal surface with the incisions carefully planned for closure to not occur over the terminal residuum.

For distal forearm or wrist-level amputations, TMR nerve transfers can be performed through a single proximal volar forearm incision. 69 To access the donor motor nerves and their recipients, an incision is made proximal to the elbow flexion crease and ulnar to the brachioradialis (BR) that extends in a curvilinear fashion along the volar mobile wad to the midportion of the distal remaining forearm. 69 Dissecting ulnar to the radial vessels, the MN is found in between the pronator teres and the FCR, and the motor branches to the palmaris longus (PL; targets) are found along the superficial surface of the flexor digitorum superficialis (FDS) deep to the PL. Alternatively, if the patient does not have a PL, the MN can be coapted to a local motor branch to the FCR or the anterior interosseous nerve (AIN). The MN may be transferred to the BR or FDS or flexor digitorum profundus (FDP) if needed, however, the motor branch to FDS is recommended to be spared as it is useful for finger flexion/grasp control. 69

The UN is transferred to a branch of the flexor carpi ulnaris (FCU) located proximally within 5 to 7 cm of the medial epicondyle. First, the UN is found in the interval between the proximal two heads of the FCU. Motor branches to FCU are locally identified and selected to receive the UN transfer. 69 The UN can also possibly be transferred to a brachialis motor branch proximal to the elbow.

After dissecting through the volar forearm fascia, dissection radial to the radial vessels will help to identify the radial sensory nerve (RSN). The RSN can be transferred to the FCR motor branch. An alternative transfer is the RSN to the BR-motor branch through a separate proximal anterolateral incision used to expose the interval between the brachialis and BR. 69 In this interval, several motor branches to the BR are found which can be coapted to the RSN.

In very distal transradial or wrist-level amputees, the sensory branches of the RN (SRN) can be transferred to the AIN to the pronator quadratus, if it remains, or the FDS, FDP or extensor carpi radialis longus. Myodesis and myoplasty are then performed to reestablish physiologic tension on the muscles and provide soft tissue coverage of the residual limb. 77 78

### Above-Knee Amputation

TMR in the setting of an above-knee amputation (AKA) requires two incisions for the transfer of the sciatic nerve (SN) and the femoral nerve (FN). The longitudinal 10-cm incision is made along the proximal third of the posterior thigh with subsequent blunt dissection revealing the hamstring musculature and SN. The SN is typically found caudal to the gluteus maximus and deep and medial to the biceps femoris. The SN is further bluntly dissected into its common peroneal and tibial components. The motor nerves of the semitendinosus, semimembranosus, and biceps femoris are then identified as the recipient motor nerves. Cadaveric studies guide the dissection with expected semimembranosus motor nerve entry approximately 0 to 50% along the length of the total thigh and long head of the biceps femoris 20 to 40%. 83 Some motor nerves leave the SN from under the gluteal muscles to innervate the proximal hamstrings. These nerves are typically 2 mm in diameter. The SN proper can be expected to give two to three motor nerve branches to each. The tibial division is transferred to the semitendinosus/semimembranosus while the common peroneal nerve is transferred to the long head of the biceps femoris. For neuroma prevention, the posterior cutaneous nerve of the thigh can be transferred to a smaller, more distal biceps femoris motor branch. 83

With the patient flipped to the supine position, a 10-cm incision paralleling and medial to the sartorius muscle is made to identify the femoral nerve in the proximal thigh. Multiple saphenous sensory nerve branches can be identified exiting immediately out of the femoral triangle, but more commonly, deeper, paralleling the femoral artery. Multiple motor branches to the sartorius and the quadriceps are available with this incision. Alternatively, the second incision can be made more medially over Hunter's canal to identify and transfer the saphenous nerve to the motor nerve of the vastus medialis. 78 84 85

### Below-knee Amputation

**Video 2** Below-knee amputation.


**Fig. 2:**
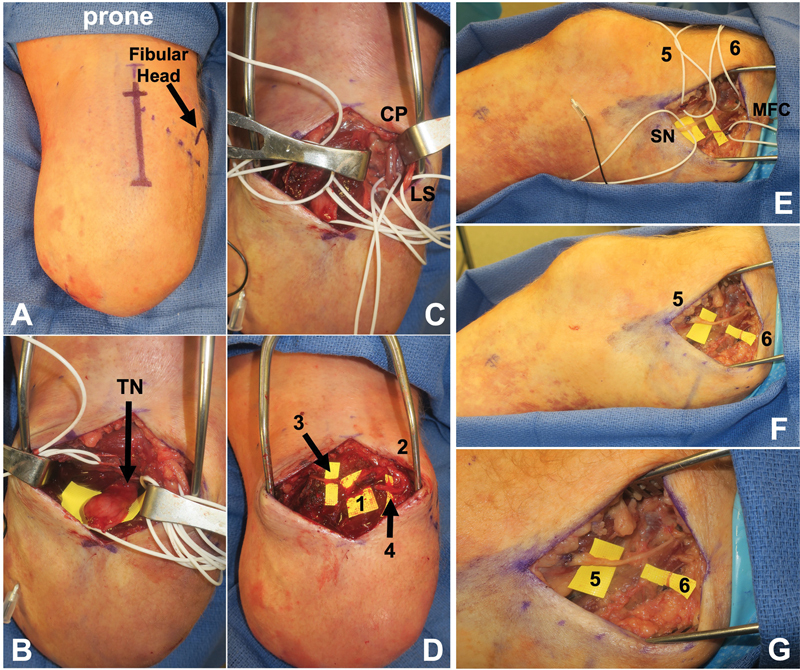
Example of delayed BKA-TMR performed via a prone and supine approach. The patient is a 45-year-old man status post-right traumatic BKA 4 years prior who presented with symptomatic neuromas and phantom limb pain. Nerve transfers were first performed in prone positioning through the popliteal fossa. ( **A** ) displays the patient in the prone position with a planned incision using the fibular head as a guide. ( **B** ) displays the TN dissected with vessel loops around motor nerve targets. ( **C** ) displays the CPN dissected out with LSN. ( **D** ) displays the coapted nerve transfers (1) TN to the motor branch of FHL, (2) CPN to the motor branch of soleus, (3) MSN to the motor branch medial gastrocnemius, and (4) LSN to the motor branch lateral gastrocnemius. ( **E** ) Displays the patient now positioned supine with motor and sensory nerve targets dissected. Video 2 displays the nerve stimulator on the motor targets. ( **F, G** ) Display the coapted nerve transfers (5) SN to motor branch vastus medialis, (6) MFCN to motor branch to sartorius. BKA, below-knee amputation; CPN, common peroneal nerve; FHL, flexor hallucis longus; LSN, lateral sural nerve; MFCN, medial femoral cutaneous nerve; MSN, medial sural nerve; SN, saphenous nerve; TMR, targeted muscle reinnervation; TN, tibial nerve.

Acute TMR in the setting of a BKA is performed in the supine position. The number of nerves transferred for acute BKA-TMR is debatable, some transfer all nerves, while others just treat the tibial (TN) and superficial peroneal nerves (SPN). The TN is typically transferred to a deep compartment motor nerve such as the flexor digitorum longus (FDL) or the soleus. The deep peroneal nerve (DPN) is preferably transferred to a tibialis anterior motor nerve, or extensor digitorum longus (EDL). The SPN is transferred to a peroneus longus motor nerve. Cadaveric studies also guide this dissection with lengths reported as measured from the lateral femoral condyle to the lateral malleolus. The FDL can be expected to have approximately six motor entry points along 30 to 90% of the leg length, and the soleus has a reliable motor entry point at 30 to 40% of the leg length. The tibialis anterior has a reliable motor entry point at 30 to 70% of the leg length, and the EDL can be expected to have approximately three motor entry points along 20 to 80% of the leg length. Finally, the peroneus longus can be expected to have five to six motor entry points along 20 to 70% of the leg length. The medial and lateral sural nerves can be coapted to motor nerves to the gastrocnemius to reduce the probability of neuroma formation, although this can often result in extensive posterior skin flap dissection. 78 83 An alternative to performing acute TMR from within the wound is to flip the patient to the prone position and to perform TMR in the posterior knee as is done for established BKA amputees ( Fig. 2 and Video 2 ). 7 83

Concerns regarding potential postoperative complications following TMR at the time of BKA exist. Complications of BKAs include infection, delayed healing, heterotopic ossification, PLP, and neuroma pain. 86 A 2022 multicenter study investigating risks of acute TMR in the setting of BKA found that TMR at the time of BKA is not associated with statistically significant increased risks of major or minor complications. 87 Another study published in 2023 of 100 highly comorbid patients treated with acute TMR at the time of BKA found similarly no increased risk of postoperative complications specific to TMR. 88 However, authors have advocated for the use of separate incisions (outside of the zone of injury) during acute TMR for BKAs to decrease the risk of wound healing complications for at-risk patients. 76

## Delayed Targeted Muscle Reinnervation

Delayed TMR can be simply defined as TMR nerve transfers performed greater than 14 days following extremity amputation or peripheral nerve injury ( **see** **Table 2** **, a summary of surgical approaches and nerve transfers for TMR in the delayed setting** ).

**Table 2:** The surgical approach to the delayed amputee is described with corresponding sensory and motor target nerves for coaptation and surgical tips for a successful operation

Amputation type	Incision/Dissection	Sensory/Mixed nerves	Target motor nerves	Prosthetic function	Notes
AKA	Single posterior incision 6–8 cm inferior limit of incision is 50% thigh length (reference contralateral)	Common peroneal	Long head biceps femoris	Dorsiflexion	Posterior cutaneous nerve of thigh transferred to most proximal motor branch to reduce tension on neurorrhaphy
		Tibial	Semitendinosus	Plantarflexion	
		Posterior cutaneous nerve of thigh	Short head biceps femoris, semitendinosus	RLP/PLP prevention	
		Saphenous	Vastus lateralis	RLP/PLP prevention	
		Lateral femoral cutaneous	Vastus lateralis, rectus femoris	RLP/PLP prevention	
BKA a	Anterolateral incision 30–60% leg length	Deep peroneal	Tibialis anterior	RLP/PLP prevention b	Very proximal BKA should use single-incision approach through single posterior midline Single-incision approach increases the chances of anterior and lateral compartment atrophy—proximal transfer of common peroneal
		Superficial peroneal	Extensor digitorum longus		
	Posterior midline incision 10–40% leg length	Lateral sural	Lateral gastrocnemius		
		Medial sural	Soleus or medial gastrocnemius		
		Tibial	Soleus		
Partial hand	Guided by amputation features and Tinel's sign	Ulnar/radial digital, common digital	Redundant intrinsic	Pain control only	Deep targets for median and ulnar-derived neuromas are the respective lumbricals/interossei
		Median nerve-derived	RMBMN adductor pollicis		
		Ulnar nerve-derived	Palmaris brevis hypothenar branches		

Abbreviations: AKA, above-knee amputation; BKA, below-knee amputation; PLP, phantom limb pain; RLP, residual limb pain.

a This describes the two-incision approach to mitigate soft tissue atrophy over the residual stump.

b Prosthetics employing these myoelectric signals currently only exist in the laboratory setting.

### Below-Knee Amputation

While delayed TMR at the AKA level is very similar to acute TMR, strategies for successful nerve transfer differ between acute and chronic TMR for the BKA patient. For delayed TMR, there is a desire not to dissect the distal stump as swelling and delayed wound healing are often problematic. Instead, the tibial and sural nerve transfers are performed in the proximal posterior leg. The TN is transferred to the motor branch to the soleus muscle. The two sural nerves (from the tibial and peroneal nerves, respectively) are transferred to the motor nerve of the FHL, or a nearby redundant motor nerve that is found intramuscularly. To maintain muscle bulk, the medial gastrocnemius is often left unperturbed, but can also be a target if necessary. Treatment of the CPN presents a challenge that is debated. One simple solution is to transfer the entire CPN to the motor nerve of the lateral gastrocnemius, which risks substantial atrophy of the anterior compartment musculature. Another solution is to perform TMR in the lateral decubitus position to perform the peroneal nerve transfers to muscles in the anterolateral compartment. The saphenous nerve is treated in the thigh at Hunter's canal with a transfer to the motor nerve of the vastus medialis ( Fig. 2 and Video 2 ). 89 90

To compare these two approaches, the single-incision approach is easier with shorter operating time, but the effort to preserve the proximal motor fascicle to the anterior and lateral compartments is required for CPN transfer to minimize the denervation and atrophy of these muscle compartments. 90 The double incision approach is useful in identifying more distal motor targets for nerve transfer of DPN and SPN and preserving muscle bulk for prosthesis wear. 90 Hence, the latter approach is more commonly performed to prevent the atrophy of the residual soft tissue. A study on TMR performed in 22 patients with BKA (18 primary, 4 secondary) reported that none of the patients developed a symptomatic neuroma for a follow-up period of 18 months after the TMR surgery. 91

### Transradial

Delayed transradial TMR can be performed through a single proximal volar forearm incision outside the zone of injury, as described above in the acute transradial TMR section. All donor and target nerves are exposed from one volar incision that extends from the elbow flexion crease to the mid-aspect of the forearm residual limb along the medial border of the BR. Similar transfers of MN to PL (or FCR, AIN, or FDP), UN to FCU, and RSN to FDS, FDP, or ECRL. 69 77

### Partial Hand Amputation

**Video 3** Delayed hand thenar.


**Fig. 3:**
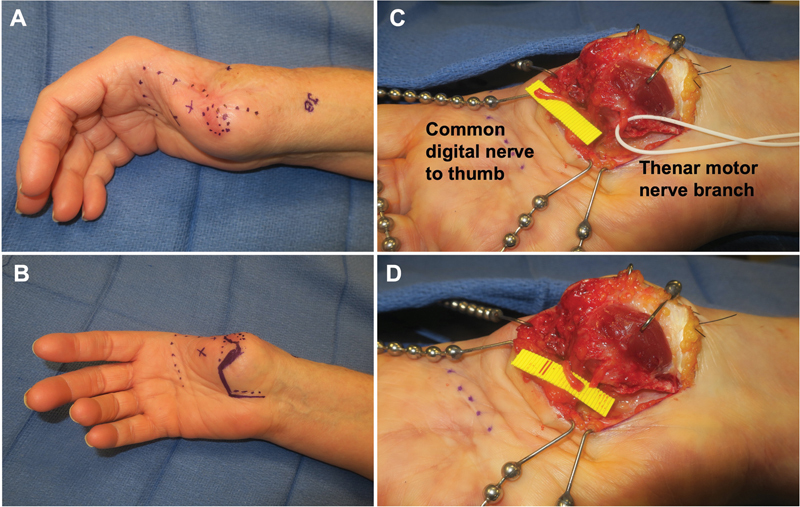
Example of delayed hand TMR. The patient is a 62-year-old female who suffered an avulsion amputation of the right thumb with failed replantation at an outside hospital, now status post multiple debridements and skin grafting with a symptomatic neuroma of the digital nerve to the thumb. ( **A, B** ) Displays the markings for the simultaneous open carpal tunnel release to easily access the thenar motor branch of the median nerve and the skin flaps that will be raised. Her first metacarpal was also noted to be prominent and painful, so this was also debrided (circular region). ( **C** ) Displays the dissected common digital nerve to thumb after distal transection to healthy fascicles and thenar motor branch (vessel loop). Video 3 demonstrates the thenar motor branch being stimulated. ( **D** ) Displays approximation of the common digital nerve to thumb and thenar motor branch just prior to coaptation. TMR, targeted muscle reinnervation.

Painful neuromas can also develop after partial hand or finger amputations, with an incidence of 7% forming after isolated digit amputations. 92 93 Case reports have demonstrated significant improvement in chronic neuroma pain in partial hand amputation patients treated with TMR. 94 Specifically, Daugherty et al 2019 presented that for ulnar/radial digital and common digital nerve painful neuromas, freshened proximal nerve endings can be successfully transferred to redundant intrinsic muscle motor nerves. 94 Cadaveric studies then demonstrated that TMR after finger or partial hand amputation is surgically feasible due to the redundant motor nerve branches to the intrinsic hand muscles. For MN-derived neuromas, superficial targets include the recurrent motor branch to the thumb, nerves to lumbricals 1/2/3, and deep targets include the adductor pollicis branches, and first and second interossei branches. For the UN-derived neuromas, targets include the hypothenar muscle branches, the third and fourth palmar and dorsal interossei, as well as lumbricals 3 and 4. 95 96 Exact TMR nerve transfers will vary depending on the specifics of the partial hand amputation ( Fig. 3 and Video 3 ).

## Chronic Neuroma Pain in the Nonamputee

TMR can be applied to treat localized chronic neuroma pain in nonamputees when other surgical interventions, such as neuroma excision and/or nerve reconstruction, have been unsuccessful or are unfeasible. A retrospective review in 2021 of 15 nonamputees who underwent TMR to redundant motor nerves for refractory neuroma pain demonstrated significant improvement in pain and quality of life. 75 Here, we describe more detailed TMR techniques for common neuromas found in the nonamputee patient population ( **Table 3** **, a summary of surgical approaches and nerve transfers for TMR in the nonamputee** ).

**Table 3:** The surgical approach to specific neuromas in nonamputees is described with corresponding sensory and motor nerves for coaptation

Neuroma	Incision location	Target motor nerves	Notes
Superficial radial	9-cm incision with distal edge 4–5 cm proximal to radial styloid	Brachioradialis Extensor carpi radialis brevis	Preceding trauma/wrist surgery
Medial antebrachial cutaneous	10 cm V-shape with apex over medial epicondyle	Flexor carpi ulnaris	Prior brachioplasty, ulnar nerve decompression, elbow surgery
Lateral antebrachial cutaneous	8 cm volar midline	Flexor carpi radialis	Prior elbow surgery
At the level of the wrist	6 cm incision proximal to carpal tunnel	Terminal AIN	Can accept dUSN, distal LABC, distal branches of the SRN, and the PCB of the median nerve
Saphenous	6–8 cm incision 10 cm cephalad to femoral condyle	Vastus medialis	Biceps femoris and gracilis are alternative targets
Medial sural	Superficial posterior 10–20% leg length	Medial/lateral gastrocnemius	Redundant innervation results in an insignificant change in function
Lateral sural		Lateral gastrocnemius	
Deep peroneal	Anterior 50–70% leg length	Extensor digitorum longus	Avoid tibialis anterior in the nonamputee—risk of foot drop
Superficial peroneal	Lateral 20–40% leg length	Peroneus longus	Redundant innervation results in insignificant change in function

Abbreviations: dUSN, dorsal sensory ulnar nerve; LABC, lateral antebrachial cutaneous; SRN, superficial branch of radial nerve; PCB, palmar cutaneous branch.

### Upper Extremity

Some of the common upper extremity painful neuromas treated with TMR include SRN neuroma, medial antebrachial cutaneous (MABC) neuroma, and LABC neuroma.

The SRN neuroma can form due to chronic compression, trauma, or wrist surgery. To locate an SRN neuroma, an incision is made over the neuroma to begin the dissection. RN neuromas may coexist with neuromatous changes of the lateral antebrachial cutaneous nerve (LABCN). Recipients for the RN transfer include the motor nerve to the pronator quadratus, ECRL, extensor carpi radialis brevis, and BR. 69 97 98 In the latter transfer, an incision is made proximal to the elbow flexion crease to locate this motor nerve.

The MABC neuroma can arise following UN decompression, elbow surgery, or brachioplasty. To approach the neuroma, a curvilinear incision is made along the course of pronator teres toward the medial epicondyle and then toward the biceps. After the neuroma excision, the MABCN can be transferred to a motor branch innervating the FCU or to a brachialis motor branch proximal to the elbow. 69

Finally, TMR for LABC neuroma involves making an 8-cm incision along the volar midline just distal to the antecubital fossa to identify the neuroma, and the transected LABCN can be transferred to a motor nerve supplying FCR. 89 A recent cadaveric study described the utility of using the terminal AIN as a recipient for TMR to treat symptomatic sensory neuromas around the wrist for more distal LABC neuromas. 99

### Saphenous

TMR is also considered for patients with unreconstructable saphenous neuromas which can develop in the medial calf after saphenous vein harvest, trauma, or vein ablation. Potential targets for an isolated saphenous nerve neuroma depend on the location of the nerve injury. While motor units to medial gastrocnemius and medial soleus muscles could potentially serve as targets, the performance of TMR at the level of Hunter's canal is less complex due to predictable anatomy, less possibility for various nerve branches, and larger nerve caliber. 83 89 90 100 When neurogenic pain occurs closer to the patella after a knee procedure, the medial approach just to the saphenous nerve at Hunter's canal may not denervate an accessory saphenous nerve branch that emerges proximal to this level. In these instances, diagnostic nerve blocks may help distinguish if there is an accessory saphenous nerve. Accessory saphenous nerve TMR is performed with an incision medial to the sartorius muscle as described in the section for TMR for the FN in AKAs.

### Sural

Sural neuromas can form after trauma, sural nerve harvest, or during exposure to foot and ankle surgery. While reconstruction using allografts has been performed in the past to restore sensation, the unpredictable relief of pain has led to the authors abandoning this approach and only performing TMR for a painful sural nerve(s). To approach the sural neuroma, a longitudinal incision is made over the Tinel's sign over the sural nerve while the patient is in the prone position. The sural nerve is characterized by its unique anatomical structure because it arises when the medial and lateral sural cutaneous nerves merge at the level of the distal third of the calf. Hence, we recommend starting the dissection from distal to proximal via a series of stairstep incisions with gentle traction on the nerve to track both medial and lateral sural cutaneous nerves. Upon making multiple incisions up to the proximal calf, a longitudinal incision is then made in the midline below the popliteal crease. The one or two sural nerves are then transected to allow for a tension-free coaptation of the newly divided motor nerve to the lateral gastrocnemius. A case report on a patient who developed sural nerve neuroma following the sural nerve graft for facial reanimation showed that the patient was pain-free with normal ambulation at a 6-month follow-up after TMR surgery. 101

## Nonextremity Painful Neuromas

### Abdominal Wall

TMR has recently been used to treat painful abdominal wall neuromas that can arise after abdominal incisions including laparoscopy. As for extremity neuromas, diagnostic nerve blocks are helpful in confirming the neurogenic origin of pain ( Table 4 ). 102

**Table 4:** Recently reported neuromas are described below for successful coaptation, each with growing bodies of literature

Neuroma	Incision location	Target motor nerves	Notes
Occipital	Longitudinal over the area of greatest tenderness	Erector spinae	Occipital nerve is traced to areas of inflamed nerve, coapted to motor branch to E. spinae
Breast	Concurrent or same incision as mastectomy	Redundant external intercostal, serratus or pectoralis	Long dissection time to locate anterior/lateral intercostal cutaneous nerves
Intercostal	8-cm incision over Tinel' sign	Internal oblique	Allograft can be used if neuroma excision limits the length Medial or lateral to semilunar line changes dissection
Ilioinguinal	2-cm incision over Tinel aimed at ASIS	Internal oblique and transversus abdominis	Motor nerve branches from ilioinguinal nerve before arises from internal oblique

Abbreviation: ASIS, anterior superior iliac spine.

### Groin—Ilioinguinal Neuroma

Chronic abdominal wall and groin pain can occur from symptomatic neuromas following surgery, massive weight loss, or abdominal wall trauma. Painful inguinal neuromas can arise after mesh inguinal hernia repair in an estimated 5% of patients. 103 104 A recent retrospective review demonstrated significant improvement in pain following TMR to treat painful ilioinguinal neuromas. 102 The reported technique is as follows: the inguinal incision of approximately one finger-breadth is made over the Tinel's sign and toward the anterior superior iliac spine. After identifying the neuroma-in-continuity of the ilioinguinal nerve below the external oblique facia, the nerve is dissected laterally to expose healthy fascicles. Then, the nerve stimulator is used to identify the 0.5 mm motor branch to the internal oblique muscle and transversus abdominis, which are typically supplied by the ilioinguinal nerve before they arise through the internal oblique muscle. This motor nerve innervates the local internal oblique muscle and is transected distally and coapted to the freshly excised donor ilioinguinal nerve ending. 102

### Intercostal Neuroma

To locate an intercostal neuroma, an 8-cm incision is made over the preoperatively marked Tinel's sign lateral or medial to the semilunar line based on physical exam and previous incisions. If making an incision lateral to the semilunar line, the intercostal nerve can be found by using a handheld nerve stimulator to identify the location of the nerve through the muscle tissues. Typically, the painful nerve can be identified by the patient in the preoperative holding area and the surgeon just needs to explore deep into the area of tenderness. For the neuroma medial to the semilunar line, the intercostal nerve arising between layers of posterior rectus fascia can be identified by making an incision through the anterior rectus fascia either in transverse or oblique fashion and mobilizing the rectus muscle medially. Upon identifying and resecting the neuroma, an interposition nerve allograft is placed. 92 Neuromas lateral to the semilunar line are neurolyzed, as often the nerves can be compressed as they travel from under or through the internal oblique fascia. A small motor nerve to the transversus abdominis is located with a nerve stimulator, and a TMR nerve transfer is performed. A retrospective study on 20 patients who underwent TMR (8 procedures), allograft nerve reconstruction (18 procedures), or both (2 procedures) demonstrated that 90% of the patients experienced a reduction in abdominal wall neuropathic pain postoperatively, with 2 patients reporting complete resolution of pain after undergoing TMR following unsuccessful nerve allograft reconstruction. 102 This retrospective study on the use of TMR in patients with abdominal wall neuromas suggests that TMR is safe and effective in reducing abdominal neuroma pain.

### Breast

Recently, TMR has been proposed as a novel surgical approach to prevent postmastectomy pain syndrome (PMPS), which affects approximately 25 to 60% of patients following mastectomy. 105 106 107 PMPS can result from symptomatic neuroma formation following mastectomy if iatrogenic injury occurs to the cutaneous intercostal nerves that provide cutaneous sensation to the breast and nipple–areolar complex. 108 109 110 111 The first step of breast TMR involves identifying all of the transected intercostal nerve branches after mastectomy. If any of the lateral cutaneous intercostal nerve branches are severed, they can be reliably found arising from the second through sixth intercostal spaces along the midaxillary line. 105 The transected anterior cutaneous nerve branches, if present, can be located lateral to the sternal border. 105 Upon identifying all the injured or transected nerves, these nerves are dissected proximally within the external intercostal muscle to increase the length of the nerve endings. 105 The nearby motor units with redundant neural input (e.g., motor units innervating adjacent intercostal muscles, serratus anterior muscle, pectoralis muscles) are identified and divided to serve as recipients for the donor transected intercostal nerves. 105 A multi-institutional case–control study on patients who underwent TMR at the time of mastectomy reported that TMR resulted in improvement in pain-related outcomes, with higher BREAST-Q physical well-being: chest scale score of 77.5 in breast surgical patients who underwent TMR in comparison to 71 in published data among patients who underwent mastectomy alone. 105 112 The study showed that after excluding the outliers, the score becomes 83.3, nearing the score of 93 in ones without a history of breast cancer or surgery. 105 112 While this study is limited by the small sample size ( *N* = 11 with 30 nerve coaptations) and a lack of a control group, the positive findings suggest the safety of using TMR as a prophylactic intervention against PMPS among breast surgical patients. 105

### Occipital Neuromas

**Video 4** Occipital nerve neuromas.


**Fig. 4:**
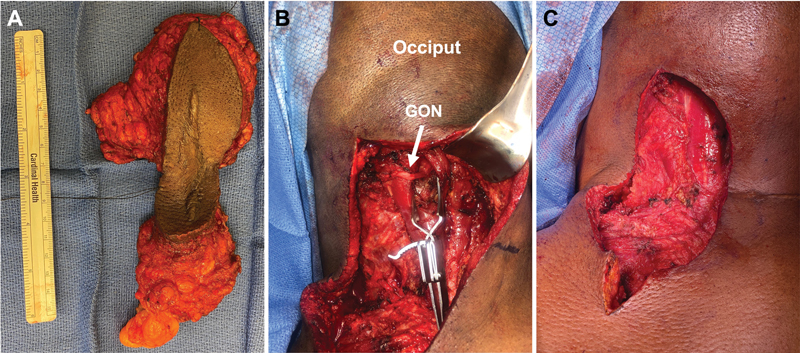
Example of acute TMR of the GON performed immediately following an oncologic resection in a middle-aged woman. ( **A** ) Demonstrates the lipomatous tumor resected by surgical oncology with ( **B** ) GON dissected and skeletonized (arrow) with the retractor assisting with visualization of the deeper motor compartments of the posterior neck for target motor nerve branches to accept the coaptation. Video 4 displays the dissection and demonstrates checkpoint stimulation of the target motor nerve to semispinalis capitis. ( **C** ) Closure of the muscle compartments after TMR. GON, greater occipital nerve; TMR, targeted muscle reinnervation.

Occipital nerve neuromas can be present after neurosurgical incisions of the cranium and after unsuccessful neurolysis or neurotomy of the occipital nerve. The refractory patient still in pain is explored in the prone position under general anesthesia. Invariably, a motor nerve to the erector spinae can be found emanating off the occipital nerve proper. After neuroma excision, TMR of the occipital nerve is performed on this small motor branch ( Fig. 4 and Video 4 ). 89

### Osseointegration

TMR combined with osseointegrated implants, or “bionic reconstruction,” is an evolving technology. 113 114 Preliminary case reports have demonstrated improved daily function with TMR performed prior to osseointegrated percutaneous implant placement and improved neuroma and PLP in upper extremity amputees. 114 115 Ongoing research is better elucidating the promise of effective osseointegrated extremity implants that can provide bidirectional motor and sensory feedback to optimize limb function in amputees. 114

### Regenerative Peripheral Nerve Interfaces

RPNI involves placing free muscle grafts over proximal transected peripheral nerve endings to serve as denervated muscle targets. The transected peripheral nerve ending then reinnervates the muscle graft. This technique has been shown, similarly to TMR, to improve myoelectric prosthesis control and to treat and prevent neuroma pain and PLP. 32 40 41 116 117 A benefit to RPNI is that it does not require a donor branch target, but a disadvantage is the small, devascularized muscle target. Although debated in the literature, a recent consensus group article stated that RPNI and TMR should be seen as “complementary” and not inferior or superior. 76 Techniques employing both TMR and RPNI have been described, which utilize free muscle grafts (RPNI) to cover TMR nerve transfers which may have a size mismatch at the coaptation site. 118 119 120 The superiority of TMR compared with TMR and RPNI remains unknown. 76

## Conclusion

In summary, TMR is an effective procedure to improve myoelectric prosthesis control and prevent and treat neuroma pain that is relatively simple to perform. Since its inception for shoulder disarticulation prosthesis control, TMR has been adapted across all major limb segments, the thoracoabdominal wall, breast, and occiput for those suffering from neuromas. The procedural techniques will continue to co-evolve with prosthetics as technology and osseointegration continue to advance for the direct benefit of amputees.
